# A cross-sectional survey of quality of life in colostomates: a report from Iran

**DOI:** 10.1186/1477-7525-10-136

**Published:** 2012-11-21

**Authors:** Bahar Mahjoubi, Rezvan Mirzaei, Rasoul Azizi, Mehdi Jafarinia, Leila Zahedi-Shoolami

**Affiliations:** 1 Colorectal Unit, Surgery Department, Hazrat e Rasoul Hospital, Tehran University of Medical Sciences, Sattarkhan Street, Niayesh Avenue, Tehran, Iran

**Keywords:** Survey, Quality of life, Colostomates, Iran

## Abstract

**Background:**

Considering the complications that colostomies may cause, patient self-assessments of their social, emotional, physical, sexual and functional conditions may help their surgeons to evaluate the impact of their interventions or use supplementary methods to maintain patient functional status or decrease its loss to the minimum level. The aim of this study was to evaluate the Quality of Life in Iranian patients with colostomies and to compare the age and gender differences among them.

**Method:**

This cross-sectional study was conducted from 2009 to 2010 to evaluate the quality of life of 96 patients who had undergone surgery for rectal cancer and had permanent colostomies. The European Organization for Research and Treatment of Cancers Quality of Life Questionnaire (EORTC QLQ)-C30 and the EORTC QLQ-CR38 were used to assess patient Quality of Life.

**Results:**

The mean scores for the functional subscales were as follows: Physical Function, 70.9 (±2.2); Role Function, 68.4 (±2.6); Emotional Function, 56.9 (±2.7); Cognitive Function, 68.7 (± 2.6); and Social Function, 64.2 (±3.3). The EORTC questionnaires showed significant differences between males and females. Males had better body image scores. Sexual Function and Sexual Enjoyment were impaired in both males and females, but males had significantly higher scores and better roles in Physical and Sexual Functions. More sexual enjoyment problems in older ages were observed in both males and females.

**Conclusion:**

Having a colostomy was associated with a high level of emotional and sexual function impairment. The differing challenges between males and females should encourage us to design sex-specific interventions that improve the quality of life in this group of patients.

## Introduction

Surgery is the initial therapy for patients with colorectal cancers [1]. Surgical exteriorization of the large bowel to the anterior abdominal wall is defined as a colostomy, which may be performed on both a permanent and a temporary basis [2].

Considering the complications that colostomy may cause and the problems from which the patient may suffer after colorectal surgeries, eliciting the patient’s self-assessment of his social, emotional, physical, sexual and functional condition before and after the operation may help the surgeon to evaluate the impact of the intervention. The surgeon can then change the treatment modalities or use supplementary methods to maintain the patient’s functional status or decrease its loss to the minimum level [3-6].

Quality of Life (QoL) is a series of subjective components reflecting aspects of patients’ physical, emotional, occupational and social experiences [7,8]. The World Health Organization (WHO) has defined QoL as “an individual’s perception of their position in life in the context of the culture and value systems in which they live and in relation to their goals, expectations, standards and concerns. It is a broad-ranging concept affected in a complex way by the person’s physical health, psychological state and level of independency, social relationships and their relationships to salient features of the environment [9]”.

Measurement of QoL provides an additional tool to assess the consequences of disease and the impact of therapeutic interventions. The European Organization for Research and Treatment of Cancers (EORTC) started a research project in 1980 to evaluate the QoL of patients with cancer and to develop core questionnaires involving a wide range of functions and modules [10].

Considering the importance of this issue, our study was designed to evaluate the Quality of Life in Iranian patients with colostomies and to analyze the results in terms of age and gender differences.

## Patients and methods

This cross-sectional study was conducted from 2009 to 2010. Patients were selected from among those who had undergone surgery and chemotherapy postsurgery for rectal cancer and who had permanent colostomies, and who also visited the colorectal clinic of the Iranian Ostomy Society (IOS) in Tehran on a routine basis. The inclusion criteria were: being an adult over 18 years of age; lack of mental impairment; and the individual’s willingness to participate in the study. The study proposal has been approved by the Ethics Committee of Hazrat e Rasoul Hospital. Ninety-six patients willing to participate in the study completed the QoL questionnaires while attending the IOS for their routine clinic visits. Sociodemographic and clinical data including age, gender, marital and occupational status and educational level were collected using patient files in the IOS. The questionnaires took at least 30 minutes to complete and have all been previously validated.The QoL aspects were assessed using the EORTC QLQ-C30 and EORTC QLQ-C38 questionnaires [11]. The EORTC-QLQ-C30 was used to assess seven aspects: functional status, role function, general symptoms, cognitive, emotional, and social functioning, and financial strain [12,13]. The EORTC QLQ-CR38 questionnaire was a site-specified module for patients with colon cancer [5]. The module comprises 38 questions assessing disease symptoms, side effects of treatment, body image, sexuality, and future perspective. All patients completed 19 questions, while the remaining questions were completed by sub samples of patients. The module was developed according to the EORTC guidelines and approved after formal review [5]. The scores ranged from 0 to 100 with higher scores indicating healthier level of functioning or a high score for a symptom scale/item representing a high level of symptomatology/problems.

### Statistical analysis

The scoring was performed using the EORTC Scoring manual [13]. Quantitative variables were described as mean (±SE) and qualitative variables as frequency (Relative frequency). Statistical significance was set as *p < 0.05*. Means, Standard Deviations (SD) and Standard Error (SE) were calculated for each of the scale measurements. Age was compared using an unpaired *t*-test. Because the QoL data were not normally distributed, nonparametric methods were used in the statistical analysis. The patient groups were compared by Chi-squared test, Mann–Whitney *U* test, Kruskal-Wallis test and Fisher’s exact test. To compare the QOL in different groups, considering the demographic variables, the One Way ANOVA test was used. SPSS® for Windows, version 11.05 was used to perform the analysis.

## Results

A total of 96 patients (42 males [43.8 percent], mean age 51.19 [range, 19–72] years; and 54 females [56.3 percent], mean age 47 [range, 27–74] years) with rectal cancer who had undergone surgery and had a permanent colostomy were entered into the study. The mean duration of follow-up in patients was 32±1.7 months post operation. The patient characteristics are displayed in Table 1. Seventy-two patients (75%) had received no information about colostomy and its complications before the surgery and 24 (25%) had received adequate information about it. Stoma-related problems were significantly lower in patients who had received adequate information about the stoma prior to surgery (34.1±4.2 vs. 58.3±3.0; *p value* = 0.00). Marital status did not change after surgery for any patient, remaining the same as it was prior to the operation. However, patient occupational status changed significantly after the surgery (*p* = 0.0). Among 54 completely active patients in our study before the operation, only 10 (18.5%) remained completely active after surgery, while 12 (22.2%) were retired and 12 (22.2%) were working as part-time employees. Twenty (37.1%) patients lost their jobs post operation. Among ten patients having a part-time job prior to surgery, 8 (80%) had lost their jobs afterwards. The mean scores for the QOL findings are displayed in Table 2.

**Table 1:** Sociodemographic characteristics of all enrolled patients

**Patient characteristics**	***Total***
Number of Patients	96
Male/Female ratio	42/54
Age (mean/Standard deviation)	48.8 (±1.49)
Education	
Not a high school graduate	12 (12.5%)
High school graduate	44 (46.8%)
College graduate	38 (40.4%)
Marital Status	
Married	76 (79.9)
Divorced	4 (4.2)
Widowed	6 (4.3)
Single	10 (10.4)
Occupational Status	
Full-time Job	54 (56.3)
Part-time Job	10 (10.4)
Retired	6 (6.3)
Unemployed	26 (27)

**Table 2:** Mean scores of EORTC QLQ-C30 and EORTC QLQ-CR38 scales and scores

**Subscales*total (n = 96)***	***Mean scores (± SE)***
**EORTC QLQ-C30**	
**Functional Scales**	
Physical Function	70.9 (± 2.2)
Role Function	68.4 (± 2.6)
Emotional Function	56.9 (± 2.7)
Cognitive Function	68.7 (±2.6)
Social Function	64.2 (± 3.3)
**Symptom Scales**	
Fatigue	45.1 (±2.6)
Pain	33 (± 3.0)
Nausea & Vomiting	10.06 (± 1.9)
**Single Items**	
Dyspnea	10.4 (± 2.2)
Insomnia	33.3 (± 3.4)
Appetite Loss	19.4 (± 2.5)
Constipation	15.9 (±2.4)
Diarrhea	18.7 (± 2.6)
**EORTC QLQ-CR38**	
**Functional Scales**	
Body Image	63.2 (± 3.1)
Future perspective	50.3 (± 4.02)
Sexual functioning	56.5 (± 3.9)
Sexual Enjoyment	24.1 (± 3.0)
**Symptom Scales**	
Radiation-Induced effects on micturition	26.9 (±2.5)
Chemotherapy side effects	19.4 (±2.1)
General Gastrointestinal Symptoms	35.1 (±2.5)
Stoma -related Problems	52.3 (± 2.7)
Sexual Dysfunction of Men	20±5.07
Sexual Dysfunction of Women	25±4.2
Weight Loss	23.7 (±3.1)

### Gender differences and QoL

The EORTC questionnaires showed significant differences between males and females. Males had better body image scores. Sexual Function and Sexual Enjoyment were impaired in both males and females, but males had significantly higher scores and better roles in Physical and Sexual Functions. Stoma related problems, general gastrointestinal symptoms and chemotherapy side effects were better tolerated in men, whereas the score for radiation-induced effects on micturition was lower in females (Tables 3 &4).

**Table 3:** EORTC QLQ-C30 functional and symptom scales in the male and female groups

**EORTC QLQ-C30**	**Male**	**Female**	**P Value**
**Functional Scales**			
Physical Function	76.5±3.3	66.6±2.8	0.02^*^
Role Function	72.2±3.6	65.4±3.7	0.2
Emotional Function	60.7±4.0	54.0±3.7	0.2
Cognitive Function	73.0±4.0	65.4±3.4	0.1
Social Function	66.6±4.3	62.3±4.8	0.5
**Symptom Scales**			
Fatigue	41.7±3.7	47.7±3.6	0.2
Pain	32.5±4.7	33.9±3.8	0.8
Nausea & Vomiting	11.9±3.5	8.6±2.0	0.3
**Single Items**			
Dyspnea	11.1±3.3	9.8±3.0	0.7
Insomnia	30.1±4.7	35.8±4.8	0.4
Appetite Loss	22.2±4.0	17.2±3.1	0.3
Constipation	19.0±4.4	13.5±2.5	0.2
Diarrhea	17.4±3.8	19.7±3.5	0.6

*p < 0.05 is considered significant.

**Table 4:** EORTC QLQ-CR38 functional and symptom scales in the male and female groups

**EORTC QLQ-CR38**	**Male**	**Female**	**P Value**
**Functional Scales**			
Body Image	75.1±3.4	52.7±4.7	0.00^*^
Future perspective	57.1±5.3	44.8±5.7	0.1
Sexual functioning	72.5±6.0	42.7±4.3	0.00^*^
Sexual Enjoyment	40.3±4.5	9.5±2.3	0.00^*^
**Symptom Scales**			
Radiation-Induced effects on micturition	33.3±3.7	21.2±3.2	0.01^*^
Chemotherapy side effects	13.2±2.1	25.1±3.4	0.005^*^
General Gastrointestinal Symptoms	29.5±3.0	40.2±3.8	0.03^*^
Stoma-related Problems	41.8±2.8	60.4±4.0	0.00^*^
Sexual Dysfunction of Men	20±5.07	-	-
Sexual Dysfunction of Women	-	25±4.2	-
Weight Loss	30.1±5.5	18.0±3.1	0.06

*p < 0.05 is considered significant.

### Age difference in sexuality and QoL

A moderate correlation was found between age and Role Function (*r =* 0.404; *p =* 0.003), Social Function (*r =* 0.315; *p =* 0.02) and Emotional Function (*r =* 0.286; *p =* 0.04) in females. Older females had a better body image score (*r =* 0.355; *p =* 0.01), a better future perspective (*r =* 0.512*; p* = 0.00), and fewer stoma-related problems (*r =* -0.483; *p =* 0.00). These effects were not observed in males (*p* > 0.05). More sexual enjoyment problems at older ages were observed in both males (*r =* -0.406; *p =* 0.01) and females (*r =* -0.419; *p* = 0.006). Males younger than the median age (53 years old) had higher sexual enjoyment scores (*p =* 0.00) and better emotional function scores (*p =* 0.01). Females younger than the median age (44.5 years) had experienced more stoma-related problems (*p* = 0.00), more gastrointestinal complaints (*p* = 0.01), more loss of appetite (*p* = 0.01) and more impairment in role and emotional function (*p* = 0.00 and 0.02 respectively) and in their future perspective (*p* = 0.04), but had higher scores in sexual function enjoyment (*p* = 0.004), in comparison with those older than 44.5 years (Table 5).

**Table 5:** Mean scores of EORTC QLQ-C30 and EORTC QLQ-CR38 scales and scores in female and male individuals

***Subscales***	**Females**			**Males**		
	***< 44.5 years old (n = 26)***	***≥44.5 years old (n = 26)***	***P Value***	***< 53 years old (n = 22)***	***≥53 years old (n = 20)***	***P Value***
**EORTC QLQ-C30**						
**Functional Scales**						
Physical Function	64.6±4.5	68.7±3.9	0.4	78.1±4.7	74.6±4.7	0.6
Role Function	58.9±5.7	76.9±2.7	0.00^*^	69.6±4.6	75±5.7	0.4
Emotional Function	44.8±5.2	62.1±5.4	0.02^*^	70.4±4.5	50.0±6.2	0.01^*^
Cognitive Function	61.5±5.4	66.6±4.1	0.4	75.7±6.2	70.0±5.0	0.4
Social Function	58.9±7.1	67.9±7.0	0.3	60.6±6.4	73.3±5.4	0.1
**Symptom Scales**						
Fatigue	54.7±4.4	40.1±5.8	0.05	37.3±4.5	46.6±6.1	0.2
Pain	35.8±4.3	32.0±6.8	0.6	33.3±5.8		0.8
Nausea & Vomiting	8.9±2.8	7.6±3.0	0.7	10.6±2.8	13.3±6.7	0.7
**Single Items**						
Dyspnea	5.1±2.4	15.3±5.6	0.1	9.0±4.4	13.3±5.0	0.5
Insomnia	38.4±6.3	35.8±7.6	0.7	21.2±4.6	40.0±8.2	0.05
Appetite Loss	25.6±4.6	10.2±4.0	0.01^*^	21.2±4.6	23.3±6.8	0.8
Constipation	12.8±4.1	12.8±3.2	0.9	27.2±6.8	10.0±4.8	0.05
Diarrhea	20.5±6.1	17.9±4.2	0.7	15.1±3.6	20.0±	0.5
**EORTC QLQ-CR38**						
**Functional Scales**						
Body Image	45.2±6.5	61.6±30.0	0.08	80.8±3.2	68.8±6.1	0.09
Future perspective	33.3±7.3	56.4±8.4	0.04^*^	57.5±6.2	56.6±9.0	0.9
Sexual functioning	46.6±5.8	39.7±6.3	0.4	81.8±6.8	61.1±10.0	0.08
Sexual Enjoyment	16.6±3.8	3.0±2.0	0.00^*^	51.5±5.6	25.0±5.6	0.00^*^
**Symptom Scales**						
Radiation-Induced effects on micturition	23.2±5.6	19.6±3.6	0.5	32.2±5.7	34.4±4.8	0.7
Chemotherapy side-effects	27.2±5.4	23.1±4.3	0.5	14.1±3.1	12.2±3.1	0.6
General Gastrointestinal Symptoms	50.3±5.5	31.1±4.6	0.01^*^	29.6±4.3	29.3±4.2	0.9
Stoma -related Problems	78.0±3.8	47.6±5.2	0.00^*^	41.9±3.8	41.6±4.2	0.9
Sexual Dysfunction	37.5±9.0	16.6±3.1	0.04^*^	28.5±9.7	12.5±3.5	0.1
Weight Loss	15.1±4.7	20.5±4.1	0.4	33.3±8.2	26.6±7.4	0.5

*p < 0.05 is considered significant.

## Discussion

Having a stoma negatively influences all aspects of a patient’s life. Although no significant relevancy was observed, most patients enrolled in our study had a high educational level. As educational level may have an influence on patient attitudes towards ostomy and on their adaptive mechanisms around this issue, it may interfere with the assessment results. In comparing males and females, women had more chemotherapy side effects, general gastrointestinal symptoms, and stoma-related problems while men had more complaints about radiation-induced effects on micturition. These findings were similar to the previous studies performed specifically on patients with colorectal cancers [10,14]. Stoma-related problems were better tolerated in men. The fear of leakage in addition to the sounds and smell which may come from the stoma bag seemed to be the main concerns for embarrassment of the young female patients. Conversely, stoma-related problems were significantly lower in patients who had adequate information about stomas prior to surgery, which highlights the importance of patient education about the principles of stoma management and complications before surgery in order to improve their self-esteem and make them feel better even with the stomas [15-17]. Having an ostomy was associated with a high level of emotional and sexual function impairment in both genders, more prominent in females. Despite having sexual dysfunction, males had better sexual function scores. Schmidt et al. reported that both genders had impaired sexual lives; however, males had significantly higher values and felt more distressed by this impairment. In males, sexuality was impaired independent of age. [10] The results of our study revealed that age was significantly correlated with sexual enjoyment impairments and that older participants had more problems with this issue. Women with colostomies reported lower physical and role function than males. They also experienced worse body images in comparison to their male peers. These were consistent with the study of Hendren et. al. which demonstrated that men had better body image scores and higher physical functions [18]. In the female groups, the older participants had higher body image scores and better emotional and role functions. They also imagined a better future perspective for themselves. It seems that older participants viewed the disease and its complications as one of the components of the aging process [18-21]. The limitations of this study merit discussion. As the study had a cross-sectional design, all patients were assessed during a limited period, which may have influenced the results of our assessments. Patients with a more prolonged history of having colostomies may have had better functional scores in comparison with those having more recent operations or being newly presented with colostomies. Covnersely, there was no valid data about patient preoperative sexual function status; therefore it is uncertain to what extent the correlations of sexual function impairments represent the influence of surgical complications or ostomy-related problems.

## Conclusion

Having an ostomy was associated with a high level of emotional and sexual function impairment and it seems that QoL assessments should be included in patient treatment protocols [1]. The differing challenges for males and females should encourage us to design sex-specific interventions to improve the quality of life in this group of patients.

## Competing interests

The authors declare that they have no competing interests.

## Authors’ contributions

BM, RM and RA carried out the study design and QoL studies while revising the article critically for important intellectual content. Mehdi Jafarinia participated in the acquisition and interpretation of data. LZ-S participated in the design of the study and performed the statistical analysis. She also participated in coordination and drafting of the manuscript. All authors read and approved the final manuscript.
